# Supplementary material for: Exploring the Therapeutic Effects of Atractylodes macrocephala Koidz against Human Gastric Cancer
Source: Nutrients. 2024 Mar 27;16(7):965. doi: 10.3390/nu16070965 (PMC11013299; doi:10.3390/nu16070965)
Supplement: Supplementary file 1 [file nutrients-16-00965-s001.zip › nutrients-2923759-supplementary.pdf]

Supplementary Material

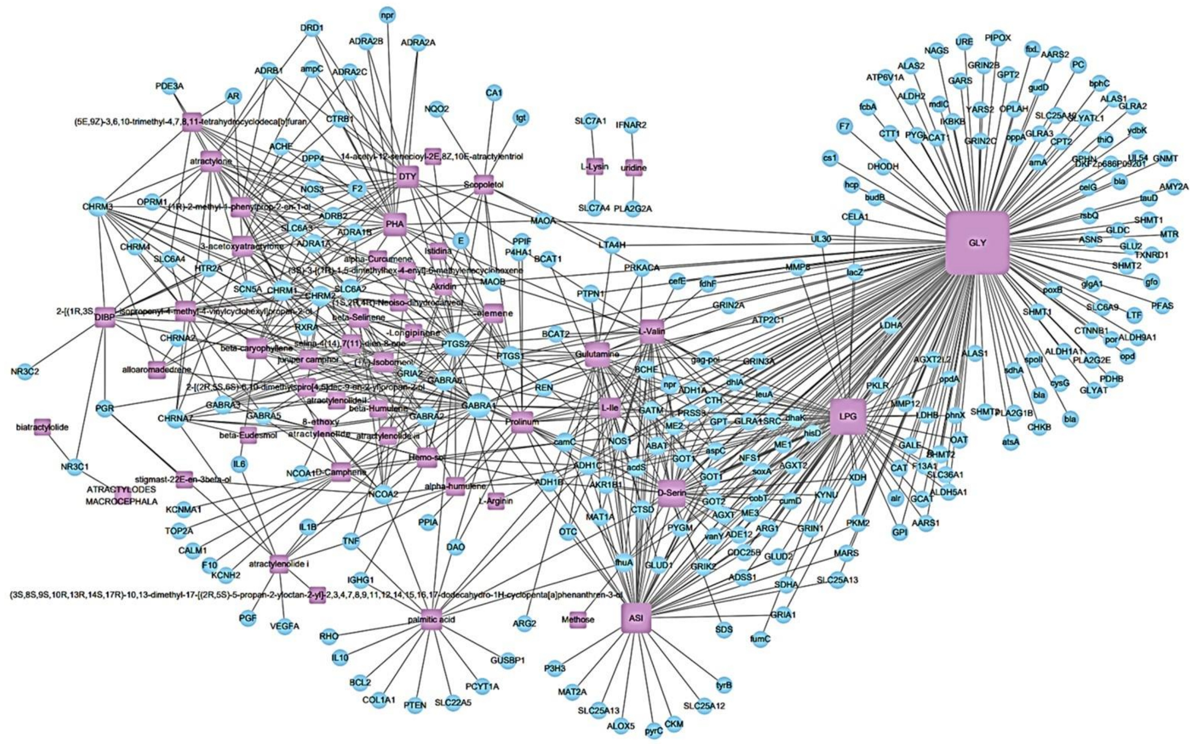

Figure S1. AMK's compound-target network. Node size in the network is determined by the number of connected edges with compounds depicted as purple square nodes and targets as blue circular nodes.

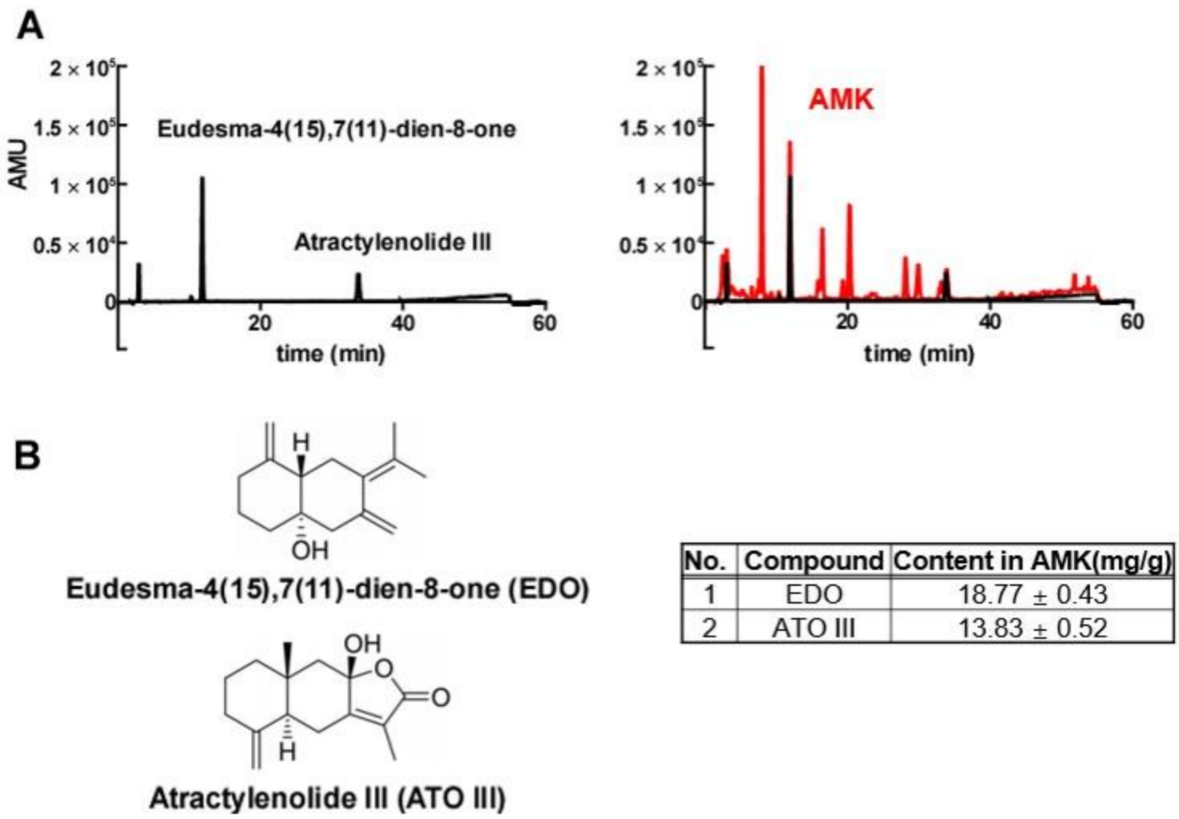

No.	Compound	Content in AMK(mg/g)
1	EDO	18.77 ± 0.43
2	ATO III	13.83 ± 0.52

Figure S2. Identification of major components of AMK by HPLC-UV. (A) HPLC-UV chromatograms of eudesma-4(15),7(11)-dien-8-one (EDO) and atractylenolide III (ATO III) in standard solution (left) and CTE (right). (B) Structural formula and contents of EDO and ATO III in AMK.

Supplementary Material Table S1.

Potential active compounds of AMK

Molecule Name	Structure	MW	OB (%)	Caco-2	DL
(+/-)-Isoborneol	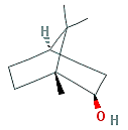	154.28	86.98	1.27	0.05
D-Camphene	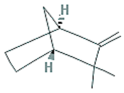	136.26	34.98	1.81	0.04

Molecule Name	Structure	MW	OB (%)	Caco-2	DL
12-senecioyl-2E,8E,10E-atractylentriol	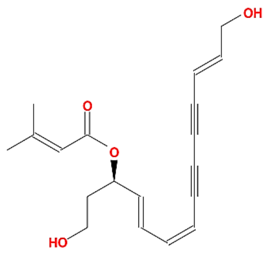	312.39	62.4	0.01	0.22
14-acetyl-12-senecioyl-2E,8E,10E-atractylentriol	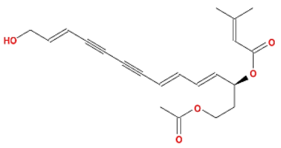	355.44	60.31	0.33	0.31
14-acetyl-12-senecioyl-2E,8Z,10E-atractylentriol	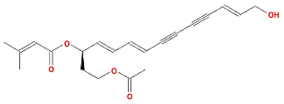	356.45	63.37	0.42	0.3
Hemo-sol	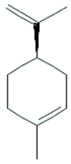	136.26	39.84	1.83	0.02
alpha-humulene	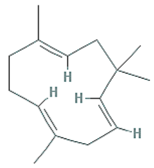	204.39	22.98	1.88	0.06
α-Longipinene	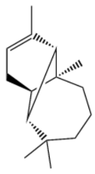	204.39	53.26	1.83	0.12
stigmast-22E-en-3beta-ol	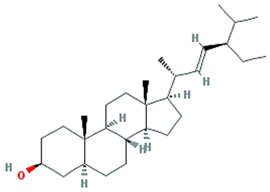	414.79	10.39	1.43	0.75

Molecule Name	Structure	MW	OB (%)	Caco-2	DL
alpha-Curcumene	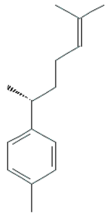	202.37	4.68	1.93	0.06
α-Amyrin	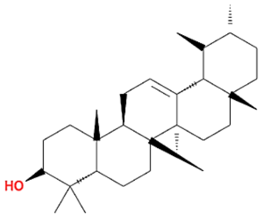	426.8	39.51	1.42	0.76
beta-Humulene	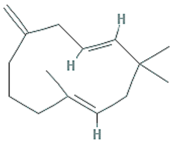	204.39	26.87	1.82	0.06
(1R)-2-methyl-1-phenylprop-2-en-1-ol	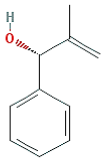	148.22	75.1	1.27	0.03
(3S)-3-[(1R)-1,5-dimethylhex-4-enyl]-6-methylenecyclohexene	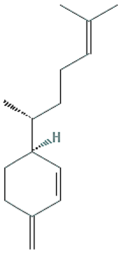	204.39	19.86	1.88	0.06
beta-Eudesmol	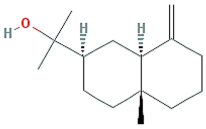	222.41	26.09	1.32	0.1
(3S,8S,9S,10R,13R,14S,17R)-10,13-dimethyl-17-[(2R,5S)-5-propan-2-yloctan-2-yl]-2,3,4,7,8,9,11,12,14,15,16,17-dodecahydro-1H-cyclopenta[a]phenanthren-3-ol	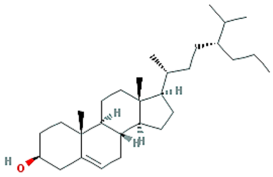	428.82	36.23	1.45	0.78

Molecule Name	Structure	MW	OB (%)	Caco-2	DL
2-[(1R,3S,4S)-3-isopropenyl-4-methyl-4-vinylcyclohexyl]propan-2-ol	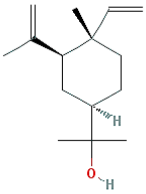	222.41	19.03	1.37	0.07
beta-Selinene	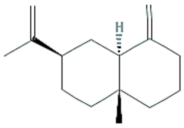	204.39	24.39	1.83	0.08
beta-caryophyllene	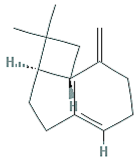	204.39	29.7	1.83	0.09
γ-elemene	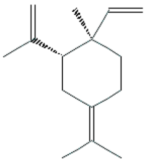	204.39	23.79	1.87	0.06
Akridin	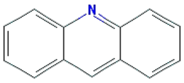	179.23	33.71	1.63	0.1
(1S,2R,4R)-Neoiso-dihydrocarveol	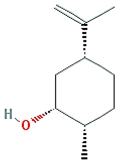	154.28	52.4	1.38	0.03
Scopoletol	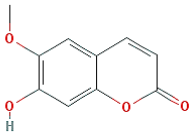	192.18	27.77	0.71	0.08
PHA	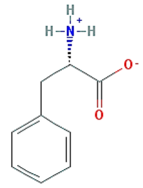	165.21	41.62	0.36	0.04
LPG	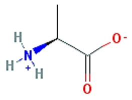	89.11	87.69	-0.34	0.01

Molecule Name	Structure	MW	OB (%)	Caco-2	DL
atractylenolide i	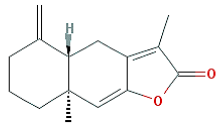	230.33	37.37	1.3	0.15
atractylenolideII	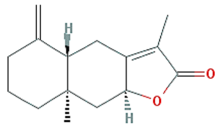	232.35	47.5	1.3	0.15
atractylenolide iii	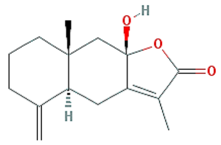	248.35	68.11	0.75	0.17
atractylone	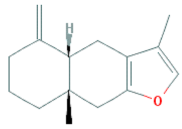	216.35	41.1	1.76	0.13
juniper camphor	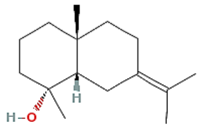	222.41	33.3	1.44	0.1
(5E,9Z)-3,6,10-trimethyl-4,7,8,11-tetrahydrocyclodeca[b]furan	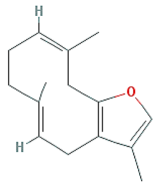	216.35	43.17	1.77	0.1
3β-acetoxyatractylone	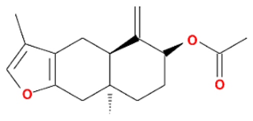	274.39	54.07	1.13	0.22
GLY	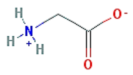	75.08	48.74	-0.56	0

Molecule Name	Structure	MW	OB (%)	Caco-2	DL
Polymannose	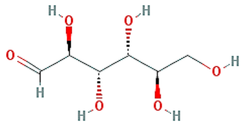	180.18	1.76	-1.94	0.03
Gulutamine	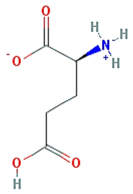	147.15	6.66	-1.05	0.02
Methose	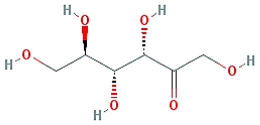	180.18	1.68	-1.8	0.03
L- Arginin	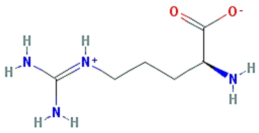	174.24	47.64	-0.49	0.03
L-Lysin	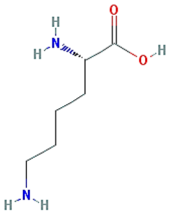	146.22	29.33	-0.66	0.02
DTY	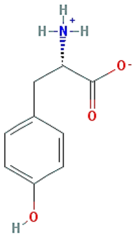	181.21	57.55	-0.1	0.05
DIBP	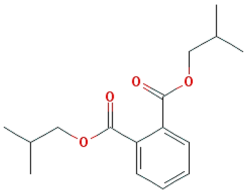	278.38	49.63	0.85	0.13
2-[(2R,5S,6S)-6,10-dimethylspiro[4.5]dec-9-en-2-yl]propan-2-ol	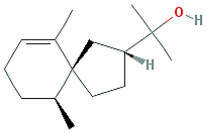	222.41	38.59	1.34	0.09

Molecule Name	Structure	MW	OB (%)	Caco-2	DL
uridine	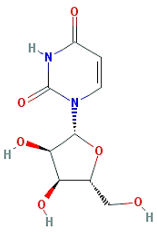	244.23	10.49	-1.14	0.11
selina-4(14),7(11)-dien-8-one	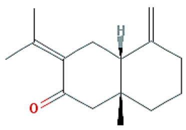	218.37	32.31	1.42	0.1
Prolinum	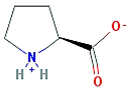	115.15	77.57	0.22	0.01
biatractylolide	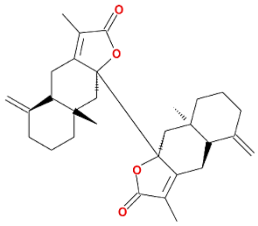	462.68	17.45	0.83	0.81
ATRACTYLODES MACROCEPHALA	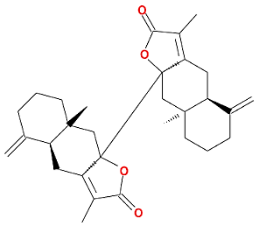	462.68	14.6	0.88	0.81
D-Serin	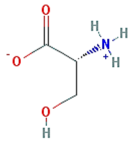	105.11	83.59	-0.94	0.01
ASI	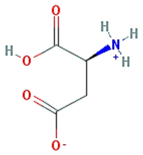	133.12	79.74	-1.02	0.02
alloaromadedrene	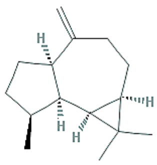	204.39	53.46	1.83	0.1

Molecule Name	Structure	MW	OB (%)	Caco-2	DL
L-Valin	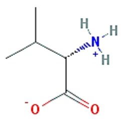	117.17	53.33	0.04	0.01
L-Ile	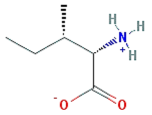	131.2	59.05	0.06	0.02
palmitic acid	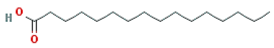	256.48	19.3	1.09	0.1
Ethyl pivaloylacetate	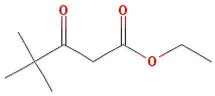	172.25	40.52	0.82	0.03
Istidina	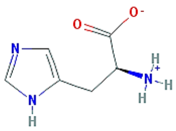	155.18	53.18	-0.25	0.03
8β-ethoxy atractylenolide Ⅲ	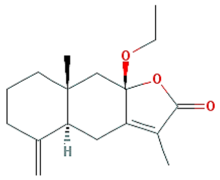	276.41	35.95	1.08	0.21

Supplementary Material Table S2. Active compounds of AMK

Molecule Name	Structure	MW	OB (%)	Caco-2	DL
12-senecioyl-2E,8E,10E-atractylentriol	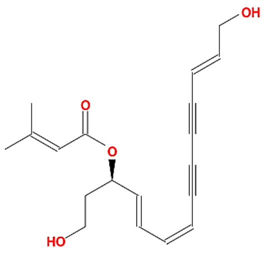	312.39	62.4	0.01	0.22
14-acetyl-12-senecioyl-2E,8E,10E-atractylentriol	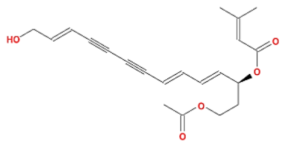	355.44	60.31	0.33	0.31

Molecule Name	Structure	MW	OB (%)	Caco-2	DL
14-acetyl-12-senecioyl-2E,8Z,10E-atractylentriol	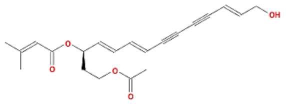	356.45	63.37	0.42	0.3
α-Longipinene	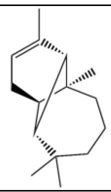	204.39	53.26	1.83	0.12
α-Amyrin	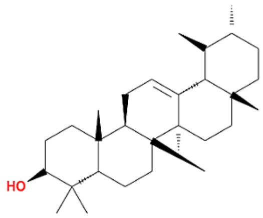	426.8	39.51	1.42	0.76
(3S,8S,9S,10R,13R,14S,17R)-10,13-dimethyl-17-[(2R,5S)-5-propan-2-yloctan-2-yl]-2,3,4,7,8,9,11,12,14,15,16,17-dodecahydro-1H-cyclopenta[a]phenanthren-3-ol	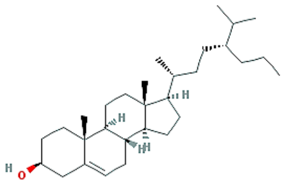	428.82	36.23	1.45	0.78
Akridin	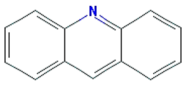	179.23	33.71	1.63	0.1
Atractylenolide Ⅰ	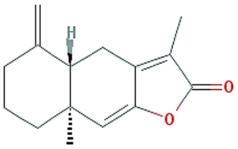	230.33	37.37	1.3	0.15
Atractylenolide Ⅱ	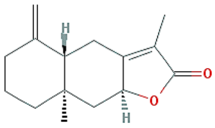	232.35	47.5	1.3	0.15
atractylenolide Ⅲ	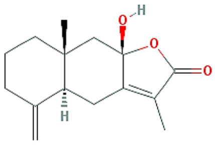	248.35	68.11	0.75	0.17
atractylone	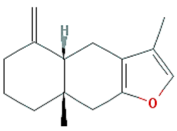	216.35	41.1	1.76	0.13

Molecule Name	Structure	MW	OB (%)	Caco-2	DL
juniper camphor	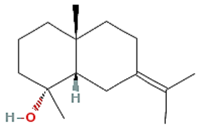	222.41	33.3	1.44	0.1
(5E,9Z)-3,6,10-trimethyl-4,7,8,11-tetrahydrocyclodeca[b]furan	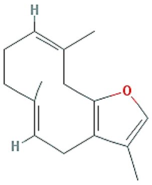	216.35	43.17	1.77	0.1
3β-acetoxyatractylone	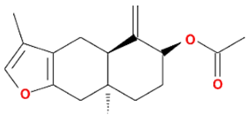	274.39	54.07	1.13	0.22
DIBP	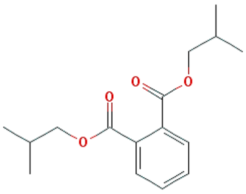	278.38	49.63	0.85	0.13
selina-4(14),7(11)-dien-8-one	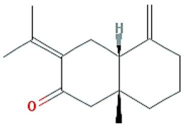	218.37	32.31	1.42	0.1
alloaromadedrene	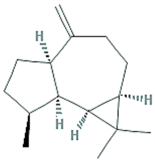	204.39	53.46	1.83	0.1
8β-ethoxy atractylenolide Ⅲ	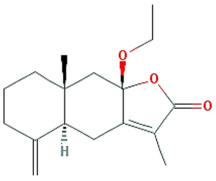	276.41	35.95	1.08	0.21
